# Treatment of Primary Mediastinal B-Cell Lymphoma With R-CEOP (Rituximab, Cyclophosphamide, Etoposide, Vincristine, and Prednisone)

**DOI:** 10.7759/cureus.16128

**Published:** 2021-07-02

**Authors:** Sundar V Cherukuri, Amit Sureen, Taylor Infante, Nevetha Rajendran, Osvaldo Padilla, Sumit Gaur

**Affiliations:** 1 Internal Medicine, Texas Tech University Health Sciences Center El Paso Paul L. Foster School of Medicine, El Paso, USA; 2 MS4, Texas Tech University Health Sciences Center El Paso Paul L. Foster School of Medicine, El Paso, USA; 3 Pathology, Texas Tech University Health Sciences Center El Paso Paul L. Foster School of Medicine, El Paso, USA; 4 Oncology, Texas Tech University Health Sciences Center El Paso Paul L. Foster School of Medicine, El Paso, USA

**Keywords:** large b cell lymphomas, reversible heart failure, r-ceop therapy, primary mediastinal b cell lymphoma, anthracyclines, non-hodgkin’s lymphomas, heart failure with reduced ejection fraction

## Abstract

Primary mediastinal B-cell lymphoma (PMBCL) is a rare subtype of non-Hodgkin's lymphoma. It typically has an aggressive behavior with potential clinical emergencies including cardiac tamponade, thrombosis of major neck vessels, airway obstruction, and tumor lysis syndrome.

In this case report, a 38-year-old Caucasian male presented with shortness of breath, a two-month history of 40-pound weight loss, and a left-sided chest wall mass. CT imaging showed a mediastinal mass, measuring 13 × 14.6 × 8.6 cm^3^, with invasion and partial occlusion of the brachiocephalic veins and upper superior vena cava causing superior vena cava syndrome, and encasement of multiple coronary artery segments. CT-guided biopsy showed high-grade B-cell lymphoma. Cytology biomarkers were positive for CD20, CD45, and PAX5. A trans-thoracic echocardiogram (TTE) was obtained prior to chemotherapy initiation to establish a baseline for cardiac function, which showed an ejection fraction (EF) of 45-50%, right ventricle volume overload and dilation, and pulmonary hypertension. R-CEOP (rituximab, cyclophosphamide, etoposide, vincristine, and prednisone) chemotherapy regimen was initiated and a follow-up echocardiogram after three cycles, revealed a significant improvement in EF; the patient subsequently received three additional cycles of R-EPOCH.

Current regimens in the United States utilize dose-adjusted R-EPOCH and R-CHOP, but they must be used cautiously in patients with compromised cardiac function, due to the cardiotoxic side effects of the chemotherapy agent, doxorubicin. This case illustrates that anthracycline-free regimens should be considered in patients with reduced cardiac function, with this case showing the utilization of an anthracycline-free regimen (R-CEOP) for the first three cycles, followed by a transition to R-EPOCH.

## Introduction

Primary mediastinal B-cell lymphoma (PMBCL) is a rare subtype of non-Hodgkin's lymphoma. The WHO 2016 revision of classification notes that PMBCL continues to be classified as a distinct entity [1]. The disease typically presents with constitutional symptoms, such as fever, weight loss, and night sweats. It often affects younger adults, with a predominance toward women. The mediastinum is the most common primary site of the disease. Tumor growth leads to infiltration of adjacent structures and leads to various symptoms, such as pleural effusions, dyspnea, dysphagia, and superior vena cava syndrome. The progression of the tumor burden dictates the onset of symptoms, typically presenting within three months [2].

Involvement of the Janus kinase-signal transducer and activator of transcription (JAK-STAT) pathway is a characteristic feature of PMBCL. It largely depends on IL-13 receptor-mediated signaling, in addition to activation from various somatic gene mutations which allow for amplification of the JAK2 molecule in the pathway. More than 50% of PMBCL cases have been shown to have gain-of-function mutations in chromosome 9p, which contains a locus for JAK2 coding [3]. There are also death ligands coded in the region as well, such as CD274 (PDL1), PDCD1LG2 (PDL2), and JMJD2C. The inactivation of these ligands offers a synergistic combination with JAK2 in PMBCL. Several other mutations can contribute to PMBCL development and progression. Point mutations in STAT6 have been found to help promote PMBCL in 36% of studied cases, and nuclear translocations of c-REL result in the activation of NF-kB, a transcription factor. Additionally, in 20% of PMBCL cases, there have been noted mutations of PTPN1, a regulator of the JAK-STAT pathway, which normally deactivates the cascade. PMBCL can evade the immune system by downregulation of MHC I and II, along with increased PDL1 and PDL2 production. This leads to immunogenicity and T-cell anergy, a feature shared with diffuse large B-cell lymphomas (DLBCL); however, the sequence of events is not yet well understood [4].

PMBCL may morphologically resemble DLBCL and may have characteristics that may overlap with gray zone lymphomas. However, DLBCL has been categorized into several subgroups based on the gene expression profiles that include germinal center B-cell (GCB), activated B-cell (ABC), and unclassified subtypes. These gene expressions include translocation of t(14;18)(q32;q21), MYC/8q24 locus, [5] *CREBBP*, *MLL2*, *BCL6*, *TP53*, and *BCL2* translocations [6]. In comparison to these DLBCL subtypes, PMBCL has been noted to have an absence in these rearrangements involving *MYC*, *BCL2*, and *BCL6*.

Histologically, PMBCL may also have overlapping morphologic features with gray zone lymphomas, but often, the clinical presentation, immunohistochemical studies, and molecular characteristics may help to differentiate between these two entities. Regardless, distinguishing between these different entities is important, due to treatment and prognostic implications.

This article was previously presented as a poster at the American Medical Student Association (AMSA) 2020 Annual Virtual Conference in April 2020.

## Case presentation

**Figure 1:**
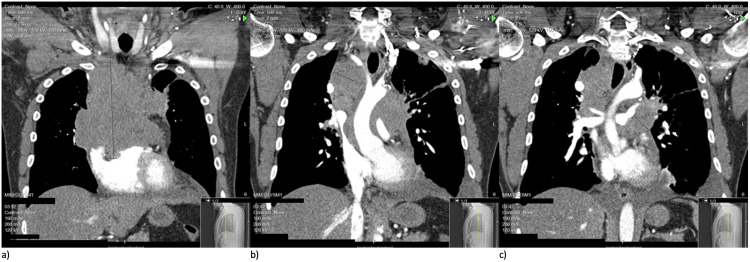
Three pre-treatment CT thorax coronal view cuts (anterior to posterior: a, b, c) depicting extensive mediastinal mass with impingement of coronary vessels, thoracic aorta, and superior vena cava. CT: computed tomography.

A 38-year-old Caucasian male presented with shortness of breath, a two-month history of 40-pound weight loss, and a left-sided chest wall mass. Over the course of one month, the mass had grown from the size of a quarter to approximately the size of an apple. One week prior to his admission, he began to experience facial swelling, flushing, non-productive cough, intermittent chest pain, and dysphagia to solid foods. He presented to our facility with a temperature of 36.7 °C, tachycardic with a heart rate of 117, respiratory rate of 18, blood pressure of 142/80 while saturating 92% on room air. Labs were significant for an absolute lymphocyte count of 0.76 µL with a lactate dehydrogenase (LDH) of 1,935. A CT scan of the thorax was performed which showed a mediastinal mass of 13 cm × 14.6 cm × 8.6 cm (TR × CC × AP) with invasion and occlusion of the brachiocephalic veins and superior vena cava. In addition, there were findings of distal superior vena cava flattening, venous collateral formation, encasement of the ascending thoracic aorta, total encasement of the right coronary artery, partial encasement of the proximal left coronary artery, and encasement and narrowing of the pulmonary artery with the possible vascular invasion of the root (Figure 1). A CT-guided biopsy was performed with biopsy results differentiating to high-grade B-cell lymphoma, favoring a DLBCL. Flow cytometry of the mass was positive for CD45, CD20, and PAX5 tumor markers and was negative for TdT, CD2, CD3, CD15, CD30, pan-keratin, and Epstein-Barr virus latent membrane protein 1 (EBV-LMP). Given the flow cytometry results and presentation, a diagnosis of PMBCL rather than DLBCL was made. Bone marrow biopsy was also obtained with no evidence of lymphoma.

A trans-thoracic echocardiogram (TTE) was performed prior to chemotherapy initiation to determine cardiac function, with results showing a reduced ejection fraction (EF) of 45-50%, right ventricular volume overload and dilation, and pulmonary hypertension. Throughout admission, the patient was persistently tachycardic requiring the use of carvedilol 25 mg BID. Due to compromised EF, R-CEOP, a non-anthracycline regimen (rituximab 375 mg/m^2^, cyclophosphamide 750 mg/m^2^, vincristine 2 mg, prednisone 100 mg PO for five days and etoposide 50 mg/m^2^ IV day 1, and 100 mg/m^2^ PO days 2 and 3), was selected as an acceptable alternative.

**Figure 2:**
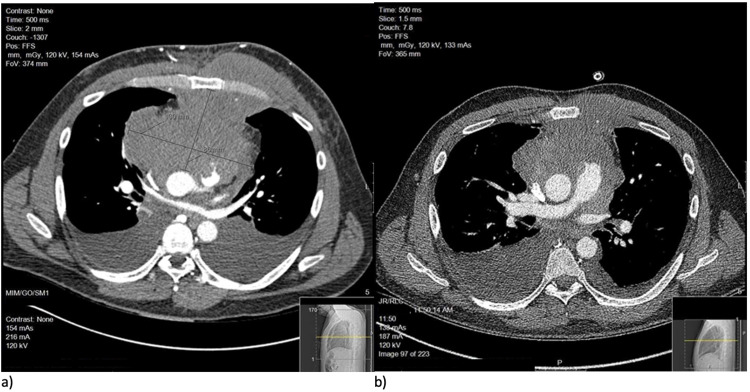
Comparative image set showcasing a pre-treatment CT thorax axial cut showing extensive mediastinal mass (a) versus CT-thorax axial cut at approximately the same level after five cycles of R-CEOP (b). CT: computed tomography.

Despite respiratory treatments, the patient developed dyspneic episodes while at rest, secondary to tumor mass effect and developing left pleural effusion. A bedside diagnostic thoracentesis was performed; yellow, hazy fluid was drawn with pleural fluid analysis suggesting an exudative effusion secondary to his malignancy. Therapeutic thoracentesis was performed the following day under ultrasound guidance and 2 L of pleural fluid was drained. A left pigtail catheter was placed to assist with continuous pleural fluid drainage. Four days later, the patient experienced a tachypneic episode, and a chest X-ray showed a moderate-sized right pleural effusion. At this time, the patient was transferred to the medical intensive care unit for closer observation. Thoracentesis was performed, with 1 L of pleural fluid removed. Sample analysis was consistent with exudative fluid, and a pigtail catheter was placed for the right effusion. Both pigtail catheters were removed approximately ten days later, toward the end of the patient’s chemotherapy cycle. Comparative CT imaging was obtained and improvement in tumor burden was noted (Figure 2).

After three cycles of R-CEOP, a transthoracic echocardiogram showed improvement in the left ventricular EF to 55-60%, at which point he was transitioned to R-EPOCH. He then completed a total of six cycles of chemotherapy. End of therapy positron emission tomography imaging noted a negative exam (Lugano score: 3 and standardized uptake value: 2.6), with no definitive quantitative scintigraphic evidence of a viable neoplasm.

## Discussion

The treatment of PMBCL largely varies. In Europe, the majority of PMBCL cases have been treated with V/MACOP-B (etoposide (VACOP-B) or methotrexate (MACOP-B), plus leucovorin (LV), doxorubicin, cyclophosphamide, vincristine, prednisone, and bleomycin). In the United States, CHOP and R-CHOP regimens have been widely used to address PMBCL cases. A retrospective study from the British Columbia Cancer Agency noted a substantial difference between regimens in five-year overall survival rate (OSR), with V/MACOP having an 87% five-year OSR, followed by R-CHOP at 82%, and lastly CHOP at 71%. In the Mabthera International Trial, three-year event-free survival was analyzed instead, which showed equal outcomes between R-CHOP and V/MACOP-B at 78% versus 52% in CHOP regimens [7]. More recent trials have reinforced the addition of rituximab to base regimens, with R-CHOP and R-EPOCH regimen variants showing improved treatment outcomes of PMBCL with an overall survival rate of 82.7-97 months [8].

Additionally, radiotherapy is often utilized in conjunction with R-CHOP and V/MACOP regimens in the treatment of PMBCL. However, to reduce the amount of overall radiation exposure to patients, investigations have been conducted recently to analyze the efficacy of dose-intensive regimens without radiation. The National Cancer Institute conducted a phase 2 trial of DA-EPOCH-R without radiation and noted a five-year event-free survival rate (EFS) of 93% [4]. Additionally, community-based trials, such as the one conducted at Guilino-Roth’s facility, have noted relatively high EFS, with their trial noting a three-year EFS of 87% [7]; however, additional trials are needed for further conclusions. Given these outcomes, radiotherapy-free regimens, such as R-EPOCH, are quickly emerging as the treatment of choice for PMBCL in the United States.

R-CHOP and R-EPOCH must be used cautiously in patients with compromised cardiac function, due to the cardiotoxic side effects of the chemotherapy agent, doxorubicin [9]. In our case, the compression of the superior vena cava resulted in SVC syndrome, and the compression of the coronary arteries caused impairment in cardiac function, in the form of reduced cardiac EF and systolic compromise. Due to this, R-CEOP, a regimen widely considered to be equivalent to R-CHOP for large B-cell lymphomas was initially selected. Studies in other cancer variations, like DLBCL, have shown comparable treatment outcomes with no statistical difference when compared to treatment with R-CHOP therapy alone [10]. However, other studies have also shown longer three-year failure-free survival times when using R-CHOP over R-CEOP [11]. R-CEOP proved to be beneficial for our case, as the tumor burden decreased, and systolic function improved after three cycles. As this was followed by three cycles of an anthracycline regimen, it is interesting to note that one study showed similar five-year OSR for patients who were treated with a combination of R-CHOP and R-CEOP compared to patients who were treated with R-CEOP alone [10].

New therapies are currently in development to further increase survival rates and event-free time periods. These medications are being tested for PMBCL cases that target the PD1 and PD2 genes located on chromosome 9p24. Pembrolizumab is one such drug candidate and has shown a 41% response rate in patients who have relapsed with PMBCL. Additionally, in all DLBCL variants, modified T-cells are being studied for activity against CD19 with the strategy to specifically target the JAK-STAT pathway or PDL activity [4]. Brentuximab vedotin, an anti-CD30 antibody-drug conjugate, has also shown a reduction in tumor volume in patients with B-cell lymphomas that contained CD30 expression. Overall response rates (ORR) of over 75% have been shown in cases of DLBCL and classical Hodgkin lymphoma, and a recent phase I/II multicenter trial showed a two-year progression-free survival rate of 86% when combined with R-CHOP [12,13]. Current findings from this trial indicate that it may be a feasible regimen against CD30 expressive lymphomas. However, other studies have noted that in relapsed PMBCL cases, there is a low ORR of 17%, with findings suggestive of resistance factors despite the presence of CD30 availability [5,14].

## Conclusions

Clinicians may consider anthracycline-free regimens if reduced cardiac function is due to organic causes. Although starting an anthracycline-based regimen to decrease the size of the large lymphoma should result in a subsequent improvement in cardiac function, our case report shows the utilization of an anthracycline-free regimen (R-CEOP) for the first three cycles, followed by a transition to R-EPOCH once the patient’s heart failure improved. A positron emission tomography scan performed at the end-of-therapy revealed no definitive quantitative scintigraphic evidence of a viable neoplasm. Overall, studies have shown similar five-year OSR for patients who were treated with a combination of anthracycline and anthracycline-free regimens compared to patients who were treated with R-CEOP alone and further studies can be performed to evaluate this efficacy.
